# Underdog Environmental Expectations and Environmental Organizational Citizenship Behavior in the Hotel Industry: Mediation of Desire to Prove Others Wrong and Individual Green Values as a Moderator

**DOI:** 10.3390/ijerph19159501

**Published:** 2022-08-02

**Authors:** Ibrahim A. Elshaer, Alaa M. S. Azazz, Sameh Fayyad

**Affiliations:** 1 Department of Management, College of Business Administration, King Faisal University, Al-Ahsaa 380, Saudi Arabia; 2 Hotel Studies Department, Faculty of Tourism and Hotels, Suez Canal University, Ismailia 41522, Egypt; sameh.fayyad@tourism.suez.edu.eg; 3 Department of Tourism and Hospitality, Arts College, King Faisal University, Al-Ahsaa 380, Saudi Arabia; 4 Tourism Studies Department, Faculty of Tourism and Hotels, Suez Canal University, Ismailia 41522, Egypt

**Keywords:** hotels industry, underdog, organizational citizenship behaviors for the environment, green values, the desire to prove others wrong

## Abstract

Discretionary environmental behaviors are usually encouraged beyond a formal reward system, but environmental skeptics, from managers or co-workers, place underdog expectations on the importance of organizational citizenship behaviors for workplace environments. Building on the leadership substitution theory, the social exchange theory (SET), and the ability-motivation-opportunity (AMO) theory, in the current study, we explore the relationships between underdog environmental expectations and organizational citizenship behaviors for the environment (OCBE), with the mediating effects of desire to prove others wrong and the moderating effect of green values. A total of 246 hotel employees participated, and the obtained data were analyzed by structural equation modeling with partial least squares (PLS). The results assert that underdog environmental expectations are able to reduce OCBE. The results also demonstrate that green values and the desire to prove others wrong lessen the negative effect of underdog environmental expectations on OCBE. In addition, we discuss the theoretical and practical implications regarding the application of these findings to the tourism and hospitality industries.

## 1. Introduction

In the late 1990s, the adoption of green practices in hotel operations became a priority [1]. The tourism sector has participated in implementing green practices due to various motivators, including financial incentives, environmental responsibility, competitiveness, legal obligations, and mounting political and institutional pressures [2,3], thus, building attractive images [4], increasing the quality of tourist products, and attracting new markets [5]. In addition, customers are willing to pay more to stay at a green hotel, and they are ready to accept minor inconveniences to participate in green initiatives due to their environmentally responsible behaviors [6]. Despite these benefits, the implementation of green practices has not been as successful as required after such efforts. Few green practice initiatives have been suitably implemented, as several obstacles impede their smooth implementation [7].

For example, some of the reasons include lack of conviction in the importance of tackling environmental issues, uncertainty about the benefits of participating in green programs and practices, and low environmental sustainability awareness and comprehension [8]. In addition, significant barriers to greening hotels include a lack of knowledge, skills, and resources; implementation and maintenance costs; and lack of hotel employees’ incentives to implement green practices [9]. As a result of these barriers, especially the absence of supervisory support for employees and the lack of conviction by the top management of the importance of environmental issues, underdog environmental expectations have been given for employees’ environmental performances, and this has been getting worse in developing nations that face dangerous environmental risks resulting from the lack of knowledge, lower interest, unavailability of sustainable materials, inefficient regulations, and the absence of incentives [10].

Employees’ voluntary pro-environmental behaviors are becoming crucial for all organizations to tackle environmental matters, including the hospitality industry; hence, there is an urgent need to understand and shape these behaviors [11]. According to [12], the spontaneity of an eco-friendly behavior or OCBE can enhance environmental performance by supplementing environmental management systems and merging environmental policies with workplace practices. In [13], the authors indicated that most studies had ignored the role of employees’ OCBE, which contributed to organizations’ environmental performances and also filled the environmental gap outside establishments’ formal systems. Based on the social exchange theory, employees are more likely to show OCBE if they feel supported and vis versa [14]. Consequently, underdog environmental expectations can negatively affect OCBE. Nevertheless, drawing on the leadership substitution theory, employees’ green values and desires to prove others wrong regarding environmental matters may substitute for supervisory support in instigating OCBE [15].

The literature on OCBE or its antecedents is still sparse and very limited [16]. In addition, the research on OCBE tends to settle that employees’ environmental concerns, supervisory support for pro-environmental behavior, and organizational pro-environmental practices are direct and independent antecedents of OCBE [15]. On the contrary, a study by [17] showed a negative relationship between supervisory support and OCBE. Therefore, environmental supervisory support may be absent, and it may even turn into skepticism about the feasibility of OCBE by setting underdog expectations for pro-environmental employees. On the basis of the leadership substitution theory, the social exchange theory, and the ability-motivation-opportunity (AMO) theory, this study seeks to test the relationship between underdog environmental expectations and OCBE, with the mediating effects of the desire to prove others wrong and the moderating effect of green values between underdog environmental expectations and the role of the desire to prove others wrong.

## 2. Theoretical Background and Development of the Hypotheses

### 2.1. Organizational Citizenship Behavior for the Environment

The concept of organizational citizenship behavior for the environment (OCBE) was first offered by Boiral in 2009, and it has since attracted the attention of many scholars [13]. OCBE evolved from the concept of organizational citizenship behaviors (OCBs) [18]. Ref. [19] defined OCB as “individual behavior that is discretionary, not directly or explicitly recognized by the formal reward system, and in the aggregate promotes the efficient and effective functioning of an organization”. Whereas, according to [20], OCBE was defined as “individual and discretionary social behaviors not explicitly acknowledged by a formal reward system and contributing to improve the effectiveness of environmental management of associations”. It is noted that the two concepts are conceptually similar in that they both emphasize volunteer behavior beyond an individual’s work functions [21]. The difference between these two concepts is that when employees perform OCBs, they target helping the organization (OCBO) and improving interpersonal relationships within the organization (OCBI), while they perform OCBE because they have an interest in the environment [22,23].

In his study, Boiral [20] depended on studying OCBE based on the six main categories of OCBs proposed by [19]: helping, sportsmanship, organizational loyalty, organizational compliance, individual initiative, and self-development. However, ref. [24] confirmed that these proposed categories were exploratory at best and relatively incomplete and required empirical validation. This was the main objective of [24], in which the authors identified three main types of OCBE, on which many studies have relied: The first is eco-helping, defined as reciprocal assistance in environmental issues, such as assisting coworkers in considering environmental concerns, sharing their opinions on the issue, and engaging in more ecologically responsible behavior; the second is eco-civic engagement, which occurs when an employee willingly participates in environmental events organized by the company, promotes the organization’s green image, and is voluntarily involved in affairs concerning the organization’s ecological issues; and the third is eco-initiatives, which refer to employee-driven pro-environmental initiatives which include workplace environmental efforts (recycling, reducing water use, saving energy, etc.), pro-environmental proposals, volunteer initiatives aimed at lowering greenhouse gas emissions, etc. [23,24].

Voluntary pro-environmental behaviors such as OCBE remain an area of particular interest for scholars because they are crucial to the sustainable performance of organizations [25]. Although OCBE may appear to be secondary when considered individually, they are expected to have a multiplier influence on environmental performance when accumulated through time and the number of people engaged [26], thus, enhancing an organization’s environmental performance [27], while also filling gaps in the formal EMS, helping the organization to reduce environmental costs, and improving the organization’s ecological reputation [13].

### 2.2. The Desire to Prove Others Wrong as a Mediator in the Relationship between Underdog Environmental Expectations and Organizational Citizenship Behavior for the Environment (OCBE)

An underdog, on the one hand, is described as a person, brand, or organization that is at a resource disadvantage and is anticipated to lose but has the passion and desire to overcome these challenges. A top dog, on the other hand, is the one who has an abundance of resources and is more likely to win the competition [28]. The underdog is the personification of the optimistic creed, i.e., where there is a will, there is a way [29]. In management, the underdog expectations concept is common, which is defined as an individual’s perception that he or she is seen as unlikely to succeed by others [30]. According to the Golem effect, behaviors reflecting low or negative supervisory expectations generate negative results in subordinates’ performances [31]. The term Golem was used to describe the negative version of Pygmalion effects, which describes the idea that boosting a superior’s expectations for a subordinate’s performance may drive that subordinate’s performance to improve [32]. Low-performance expectations may jeopardize employees’ perceptions of their abilities and put their achievements into doubt [33], and the heighten self-doubt and anxiety disable their performances [34]. In addition, according to [35], high expectations are more likely to drive employees to quit when they are performing poorly due to the pressure of embarrassment, especially when employees themselves do not believe that they can succeed. Often, when performance expectations are high, an employee commonly decreases personal performance objectives or standards, their level of performance tends to drop off, and they are likely to develop negative attitudes toward the job [36].

Barriers related to environmental attitudes are the most significant impediments to environmental initiative implementation [37]. These barriers include negative organizational attitudes toward environmental initiatives and unfavorable corporation cultures, resistance to change within organizational cultures, fear of free-riders [38], inconsistent support from top management [37], disbelief of the benefits of environmental initiatives [39], the perceived high effort needed [40], lack of internal marketing of environmental initiative, and negative background or experience with some environmental standards that rubs off on employee environmental participation acceptance [37]. These barriers cause organizations to be skeptical about the feasibility of environmental efforts and their outcomes. Hence, underdog environmental expectations for employees’ voluntary efforts are likely to be displayed. According to the social exchange theory and the ability-motivation-opportunity (AMO) theory, the literature on OCBE tends to take it for granted that leadership and supervisory support have non-substitutable significance [15], since employees cannot be forced to enact OCBE, because it is a voluntary behavior [41]. Psychological support provided by an environmental leader helps employees to deal with “green” matters innovatively [42], and employees rely on this support as a resource to reduce uncertainty and bring about clarity related to green issues [43]. Referring to counterproductive workplace behaviors such as underdog environmental expectations, researchers have asserted that these unfavourable mechanisms can disable the desired behaviors [44]. On this basis, we develop the hypothesis:

**Hypothesis** **1** **(H1).** *Underdog environmental expectations are negatively related to OCBE*.

Neveretheless, depending on self-enhancement and psychological reactance theories, ref. [30] proposed that underdog expectations could motivate employees to perform better through a desire to prove others wrong [33]. The desire to prove others wrong refers to an individual’s motivation to demonstrate that others’ thoughts and perspectives are erroneous [30]. When employees experience low expectations, they display high resilience to seek to prove others wrong by working harder [35,45]. It can be asserted that according to the Galatea effect, employees can use these underdog expectations to raise their self-expectations regarding their performance, thus, also raising their level of performance and increasing their desire to prove others wrong [32]. Therefore, we hypothesize the following:

**Hypothesis** **2** **(H2).** *Underdog environmental expectations are positively related to the desire to prove others wrong*.

Even if supervisory supports for pro-environmental behavior and organizational pro-environmental practices are absent, pro-environmental employees, based on the “leadership substitution” theory, use their individual values and their desire to prove others wrong to neutralize the effect of leadership behavior and make leadership less relevant, to engage in OCBE [15]. Previous studies have discovered that individual values impact employees’ OCBE [20,46]. Thus, it can be said that individual environmental values stimulate the desire to prove others wrong who predict underdog environmental expectations. Based on these arguments, the following is hypothesized:

**Hypothesis** **3** **(H3).** *The desire to prove others wrong is positively related to OCBE*.

**Figure 1:**
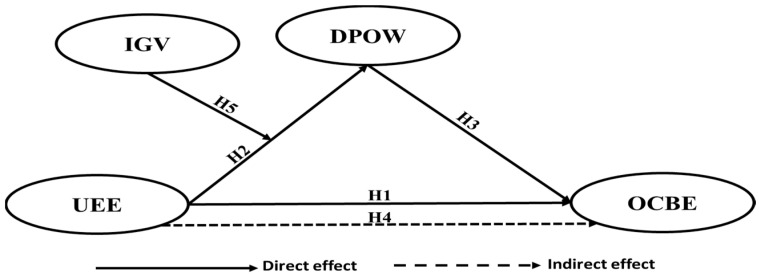
The proposed conceptual framework and hypotheses. UEE → underdog environmental expectations; OCBE → organizational citizenship behavior for the environment; DPOW → the desire to prove others wrong; IGV → individual green values.

According to the arguments presented above that explain the relationships between underdog environmental expectations and OCBE, underdog environmental expectations and the desire to prove others wrong, and the desire to prove others wrong and OCBE, the following hypothesis is proposed, as shown in Figure 1:

**Hypothesis** **4** **(H4).** *The desire to prove others wrong mediates the relationships between underdog environmental expectations and OCBE*.

### 2.3. Individual Green Values as a Moderator in the Relationship of Underdog Environmental Expectations and the Desire to Prove Others Wrong

According to the value-belief-norm (VBN) theory, personal values, beliefs, and norms affect people’s work behaviors [46]. Thus, individuals’ concerns for environmental values significantly influence their green behavior [47]. Consistent with self-determination theory, refs. [48,49] suggested that employees with assertive environmental beliefs had higher ecological commitment and were self-motivated to become more involved in OCBE. Individuals’ environmental beliefs are affected by their green values that are generated from their understanding of threats and negative environmental outcomes, and thus, affect their norms [46]. Thus, we can assume that green values boost the ability of individuals to overcome others’ underdog environmental expectations by supporting their desire to prove others wrong. Accordingly, this study suggests the following hypothesis:

**Hypothesis** **5** **(H5).** *Individual green values moderate the influence of underdog environmental expectations on the desire to prove others wrong, such that the relationship will be stronger when individual green values are high*.

## 3. Materials and Methods

This study employed a quantitative research method with a structured questionnaire as the main research tool to collect the required data. It is a popular low-cost method to collect a large sample size of a particular population. The authors started by designing the research instrument. Consequently, data were collected and analyzed with structural equation modeling (SEM) using the Smart partial least squares (PLS) program. 

### 3.1. Instrument Measurement

In order to assess the study’s hypotheses, a questionnaire was developed, and the study’s measures were identified through a comprehensive analysis of prior empirical research. Based on the results of the previous process, four dimensions emerged. The UEE and DPOW were measured using 9 items based on a study by Nurmohamed [30]. The OCBE was operationalized by using a 7-item scale suggested by Boiral and Paillé [24]. Finally, 3 items from Chen and Jin [47] were used to operationalize IGV. A Likert scale of 5 points was adopted, where 1 reflected “strongly disagree” and 5 meant “strongly agree”. The scale was validated by some academics and professional in the hotel industry with no major corrections (8). 

### 3.2. Participants and Data Collection

A total of four hundred questionnaires were handed out by the research team. Members of the research team were employed by various tourism and hotel management educational institutions. As a consequence of this, they had a good personal connection with hotel human resources managers (HRMs). HRMs assisted in collecting the required data from guest-contact employees at Sharm El-Sheikh hotels (which is located in Egypt) during the month of January 2022 using convenience sampling and drop and collect methods. Sharm El-Sheikh city was selected as it possesses numerous high ranked five-star hotels. Employees with a minimum of four years of experience were permitted to respond to the survey because they possessed sufficient experience to answer the study questions. In total, 114 out of the 400 questionnaires were excluded due to insufficient answers, leaving a total of 246 valid questionnaires with a recovery rate of 62%. Respondents were required to sign a consent form, and they were given the choice between participating in the survey or skipping it, all participants being assured that their answers would be kept confidential. The study sample consisted of 74.4% males and 25.6% females aged between 26 and 58 years old. There were fewer unmarried employees (21.5%) than married employees (78.5%). The vast majority of respondents (78%) had obtained bachelor’s degrees. Additionally, most participants (98%) were Egyptian, but only 2% had a non-Egyptian nationality (generally employees in the animations team or the public relations section). Slightly more than 50% (58%) of the participants had been working in the hotel with more than 7 years of experience, whereas 42% had from 4 to 7 years of experience.

A non-response bias analysis was carried out with the help of an independent *t*-test sample technique. Because the mean variance between late and early answers did not show any significant statistical value (*p* > 0.05), bias from non-response was not a concern in this study [50].

## 4. Results of Data Analysis

The current study employed and conducted the “structural equation modeling” (SEM) with “partial least squares” (PLS) method to test the hypotheses of the research with the SmartPLS version 3.0. program. The pre-justified conceptual model was assessed with a two-step methodology, as recommended by Leguina [50], and is described below.

### 4.1. Outer Measurement Model Assessment

To assess the outer model’s validity and reliability, convergent validity, discriminant validity, internal consistency reliability, and indicator reliability were all examined. As shown in Table 1, the reliability “structures’ internal consistencies” were examined with Cronbach’s alpha (α) ranging from 0.874 to 0.920 and the composite reliability (CR) values were found to be between 0.922 and 0.938.

Second, indicator reliability scores were satisfactory as all factor loading values of the structure variables exceeded the value of 0.60. Third, convergent validity was examined with the average variance extracted (AVE) scores, which were found to be higher than the threshold value of 0.50 [50]. Finally, three conditions were assessing to confirm the discriminant validity of the measures: cross-loading, Fornell–Larcker criterion, and the heterotrait-monotrait ratio (HTMT) [50]. As depicted in Table 2, the outer-factor loading for each latent observed variable (underlined) was higher than the cross-loading results with other variables.

As depicted in Table 3, the bolded scores of the AVEs in the diagonal line are higher than the correlation coefficients between the research variables [51]. The HTMT findings should be less than 0.90 to confirm discriminant validity [51]. The values of HTMT, as shown in Table 3, did not exceed this threshold. The findings show that the structure model has adequate discriminant validity. As a result, the outer measurement model’s findings were sufficient to proceed with the structural model assessment. 

### 4.2. Structural Model Assessment 

Next, the proposed research hypotheses were examined using a structural equation analysis (SEM). Consequently, the predictive and explanatory power of the structure model were evaluated [52]. With the VIF scores of the manifest variables varying from 1.847 to 4.085, these values are below the suggested threshold value of 5.0, giving signals for the inexistent of multicollinearity in the structural model. Chin, ref. [53] suggested that the lower value for the R2 score should be 0.10. Therefore, the R2 values for the indicators of OCBE (R2 = 0.460) and DPOW (R2 = 0.396) are adequate, as shown in Table 4. Furthermore, the Stone–Geisser Q2 assessment showed that the OCBE and DPOW scores were higher than zero (Table 4), demonstrating a satisfactory predictive power of the structural model [54]. 

**Figure 2:**
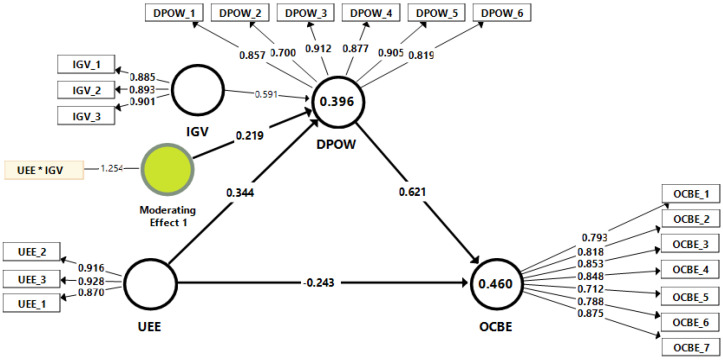
The tested structural and measurement model. UEE → underdog environmental expectations; OCBE → organizational citizenship behavior for the environment; DPOW → the desire to prove others wrong; IGV → individual green values.

Finally, the path coefficient values with their associated t-value of the hypothesized relationships were evaluated with the bootstrapping method. Figure 2 and Table 5 illustrate the findings of the analyses of the hypotheses, given the path coefficient (β) values and the relevant significance *p*-values. The UEE were found to have a negative but significant impact on OCBE at β = −0.243, *p* < 0.01, while it showed a positive and significant impact on DPOW, at β = 0.344, *p* < 0.01, thus H1 and H2 were accepted. The results of the Smart PLS demonstrated that DPOW significantly and positively impacted OCBE (β = 0.621, *p* < 0.01), confirming H3. As for the mediation effects, UEE were found to positively affect OCBE through DPOW (indirect effect) at β = 0.214, *p* < 0.01, confirming H4. Finally, the findings support the positive moderation impact of IGV on OCBE towards the DPOW at β = 0.219, *p* < 0.01, which support H5. 

## 5. Discussion and Implications

### 5.1. UEE, OCBE, and DPOW

The empirical results of the current study showed that the UEE had a negative effect on OCBE. This result agrees with the explanation of the Golem effect that asserts that low or negative expectations for employees’ behaviors generate negative results in their extra-role (i.e., OCBE) or in-role performances [31]. Furthermore, they are consistent with [33], who confirmed that underdog performance expectations put the abilities and achievements of the employees into doubt, which increased their anxiety that disabled performance [34]. Based on the social exchange theory, we can argue that employees are not more likely to display OCBE if they are given underdog expectations. The results also showed that the UEE positively affect DPOW. This agrees with self-enhancement and psychological reactance theories which argue that underdog expectations can motivate employees to perform better through a desire to prove others wrong [30]. In the same vein, the results found that the DPOW variable positively influences OCBE. It was expected that the desire to prove others wrong to refute their underdog expectations regarding environmental initiatives would positively affect OCBE, given that this proving process may include considerable voluntary environmental behaviors. Based on the “leadership substitution” theory, pro-environmental employees may use their individual values and their desire to prove others wrong beyond leadership behavior to engage in OCBE [15]. 

### 5.2. Assessing the Moderating Effect

**Figure 3:**
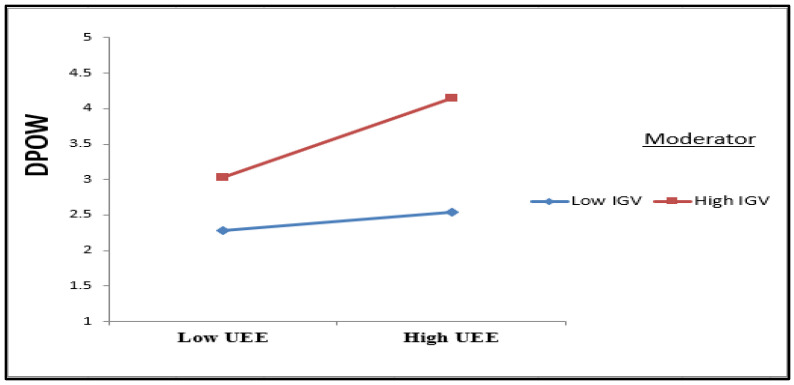
Interaction plot for the IGV moderation effect on UEE towards DPOW.

The practical results validated the positive moderation influences of the IGV variable on the relationship between UEE and DPOW. In other words, IGV can strengthen the positive relationship between UEE and DPOW (Figure 3, interaction plot). Returning to Figure 2 and calculating the moderator’s interaction values (0.344 + 0.219 = 0.563), we conclude that IGV strengthened the relationship between UEE and DPOW. This result agrees with the findings of [46], who debated that the green values of employees that are generate from their understanding of threats and negative environmental outcomes affect their behavior norms which drive them to encounter underdog environmental expectations by proving the correctness of their behaviors. Furthermore, based on self-determination theory, pro-environmental employees’ beliefs heighten ecological commitment and self-motivated to become more involved in OCBE by refuting the underdog environmental expectations of environmental skeptics [48,49].

### 5.3. The Mediating Role of DPOW between the Relationship UEE and OCBE

One of this study’s main aims was to examine the mediating role of DPOW between UEE and OCBE. The study’s findings indicated that OCBE positively and significantly mediated the relationship between UEE and OCBE. It is worth noting that the negative direct relationship between UEE and OCBE changed into a positive indirect relationship through DPOW. This result is consistent with a study by [32], who argued that, according to the Galatea effect, employees could use underdog expectations to raise their self-expectations regarding their performances, thus, also raising their level of performance and increasing their desire to prove others wrong, and finally display the desired behaviors (OCBE).

Environmental skepticism is doubt about the authenticity or severity of environmental degradation, which is widespread among the public [55]. Actually, Opin polls have suggested that environmental skepticisms have recently increased in Europe [56] and the USA [57]. It is undoubtedly more exacerbated in developing countries, which can become a significant obstacle to developing an environmentally sustainable society. Furthermore, these skepticisms can impede individuals’ engagement in environmental behaviors by placing underdog environmental expectations on ecological initiatives, especially voluntary behaviors (OCBE). Therefore, we should be concerned about the impact of skeptical attitudes on actions to tackle environmental problems. This is given that environmental skeptics also exist within organizations and may be in supervisory positions. Therefore, the current study suggests that supporting green values and beliefs is the first step toward facing environmental skepticisms and increasing the ability of pro-environmental employees to prove the environmental skeptics’ claims wrong. Thus, organizations can reap the benefits of OCBE.

## 6. Conclusions

Underdog environmental expectations were given for employees’ environmental performances due to several barriers to greening hotels initiations. Examples of some barriers include a lack of conviction in the importance of tackling environmental issues; uncertainty about the benefits of participating in green programs and practices; low environmental sustainability awareness and comprehension, a lack of knowledge, skills, and resources; high costs of implementation and maintenance; and lack of hotel employees’ incentives to implement green practices. Most previous research papers have neglected the important role of the employees’ OCBE, which not only improve the organization’s environmental performance but also fill the environmental gap outside establishments’ formal systems. According to the social exchange theory, employees are more likely to engage in OCBE practices if they gain support and vis versa. Therefore, underdog environmental expectations may have a negative impact on OCBE. Nevertheless, based on the leadership substitution theory, an employee’s green values and desire to prove others wrong regarding environmental issues may substitute for supervisory support in initiating OCBE. The current study aimed at investigating underdog environmental expectations and environmental organizational citizenship behavior with the mediation of desire to prove others wrong and individual green values as a moderator. 

Data were gathered from 264 guest-contact employees of Sharm El-Sheikh hotels. The scale’s convergent and discriminant validity and the research hypotheses were evaluated by conducting SEM with the Smart PLS program. The results showed that the scale has adequate convergent and discriminant validity. The testing of hypotheses showed results that were consistent with the social exchange theory, where employees were not more likely to exhibit OCBE when provided with underdog expectations. The study findings were also consistent with the self-enhancement theory and psychological reactance theory, both of which contend that underdog expectations can inspire employees to behave better through a want to prove others are wrong [30]. As was expected, the results found that the DPOW positively impacted OCBE, where the desire to prove others wrong led to disprove underdog expectations regarding environmental initiatives, and consequently, positively impacted OCBE. Building upon the “leadership substitution” theory, pro-environmental employees may use their personal values and desires to give evidence of others wrong, beyond leadership behavior to participate in OCBE [15]. The results also give evidence that green values (as a moderator) and the desire to prove others wrong (as a mediation) lessen the negative impacts of underdog environmental expectations on OCBE.

Our study tried to build on the leadership substitution theory by discussing the issue of OCBE as compared with most studies that have tended to take it for granted that leadership and supervisory support have non-substitutable significance, although, in supervisory positions, there may be environmental skeptics. Thus, using leadership substitution theory in the debate about OCBE is a contribution in its own right.

## 7. Limitations and Future Research Avenues

Similar to other studies on this topic, the current study has a number of limitations, and we recommend exploring additional research avenues. First, this study investigated the impact of underdog environmental expectations (UEE) on organizational citizenship behaviors for the environment (OCBE) with the mediating role of desire to prove others wrong (DPOW) and the moderating role of individual green value (IGV); however, other dimensions could be further investigated as a moderator, such as trust in supervisors, distributive justice, and stress copying style, religioisty and culture [58], while other dimensions could be further tested as mediator such as self-efficacy, job satisfaction and employee involvement [59]. Second, cross-sectional data prevent precise causal impacts between latent variables. Future scholars may use longitudinal data or multiple data sources to validate a study’s structure model before and after the COVID-19 pandemic. Fourth, a multi-group analysis method could be used to evaluate these relationships in other distinct contexts (country or industry).

## Figures and Tables

**Table 1:** Assessment of the formative measurement model.

Abbreviation	Outer Loading	α	CR	AVE
UEE		0.890	0.931	0.818
UEE_2	0.916			
UEE_3	0.928			
UEE_1	0.870			
OCBE		0.915	0.932	0.662
OCBE_1	0.793			
OCBE_2	0.818			
OCBE_3	0.853			
OCBE_4	0.848			
OCBE_5	0.712			
OCBE_6	0.788			
OCBE_7	0.875			
DPOW		0.920	0.938	0.719
DPOW_1	0.857			
DPOW_2	0.700			
DPOW_3	0.912			
DPOW_4	0.877			
DPOW_5	0.905			
DPOW_6	0.819			
IGV		0.874	0.922	0.798
IGV_1	0.885			
IGV_2	0.893			
IGV_3	0.901			

**Table 2:** Cross loading results.

Abbreviation	DPOW	IGV	OCBE	UEE
DPOW_1	0.857	0.487	0.537	−0.108
DPOW_2	0.700	0.371	0.506	−0.058
DPOW_3	0.912	0.446	0.559	0.018
DPOW_4	0.877	0.388	0.537	−0.043
DPOW_5	0.905	0.492	0.543	−0.036
DPOW_6	0.819	0.416	0.538	−0.027
IGV_1	0.514	0.885	0.458	−0.533
IGV_2	0.415	0.893	0.589	−0.433
IGV_3	0.433	0.901	0.558	−0.435
OCBE_1	0.550	0.469	0.793	−0.092
OCBE_2	0.634	0.428	0.818	−0.191
OCBE_3	0.599	0.516	0.853	−0.147
OCBE_4	0.452	0.446	0.848	−0.265
OCBE_5	0.289	0.416	0.712	−0.263
OCBE_6	0.475	0.516	0.788	−0.290
OCBE_7	0.517	0.582	0.875	−0.338
UEE_2	−0.106	−0.506	−0.266	0.916
UEE_3	−0.037	−0.480	−0.247	0.928
UEE_1	0.026	−0.442	−0.225	0.870

**Table 3:** Inter-construct correlations, the square root of AVE, and HTMT results.

	AVEs Values				HTMT Results			
	DPOW	IGV	OCBE	UEE	DPOW	IGV	OCBE	UEE
DPOW	0.848							
IGV	0.513	0.893			0.566			
OCBE	0.633	0.593	0.814		0.675	0.669		
UEE	−0.049	−0.528	−0.274	0.905	0.093	0.590	0.307	

**Table 4:** Coefficient of determination (R2) and (Q2) of the model.

Endogenous Latent Construct	(R2)	(Q2)
OCBE	0.460	0.275
DPOW	0.396	0.257

**Table 5:** The structural model’s results.

	Hypotheses	Beta (β)	T-Value	*p*-Value	Results of Hypotheses
H1	UEE → OCBE	−0.243	4.859	0.000	Accepted
H2	UEE → DPOW	0.344	5.627	0.000	Accepted
H3	DPOW → OCBE	0.621	9.757	0.000	Accepted
H4	UEE → DPOW → OCBE	0.214	4.800	0.000	Accepted
H5	Moderating effect 1 (UEE ∗ IGV) → DPOW	0.219	3.823	0.000	Accepted

## Data Availability

Data is available upon request from researchers who meet the eligibility criteria. Kindly contact the first author privately through e-mail.
